# LCCNN: a Lightweight Customized CNN-Based Distance Education App for COVID-19 Recognition

**DOI:** 10.1007/s11036-023-02185-9

**Published:** 2023-07-29

**Authors:** Jiaji Wang, Suresh Chandra Satapathy, Shuihua Wang, Yudong Zhang

**Affiliations:** 1 School of Computing and Mathematical Sciences, University of Leicester, Leicester, LE1 7RH UK; 2 School of Computer Engineering, KIIT Deemed to University, Bhubaneswar, India; 3 School of Computer Science and Technology, Henan Polytechnic University, Jiaozuo, Henan 454000 People’s Republic of China; 4 Department of Information Systems, Faculty of Computing and Information Technology, King Abdulaziz University, Jeddah, 21589 Saudi Arabia

**Keywords:** Distance education, COVID-19, Medical image diagnosis, Deep learning, Web app, Convolutional neural network

## Abstract

In the global epidemic, distance learning occupies an increasingly important place in teaching and learning because of its great potential. This paper proposes a web-based app that includes a proposed 8-layered lightweight, customized convolutional neural network (LCCNN) for COVID-19 recognition. Five-channel data augmentation is proposed and used to help the model avoid overfitting. The LCCNN achieves an accuracy of 91.78%, which is higher than the other eight state-of-the-art methods. The results show that this web-based app provides a valuable diagnostic perspective on the patients and is an excellent way to facilitate medical education. Our LCCNN model is explainable for both radiologists and distance education users. Heat maps are generated where the lesions are clearly spotted. The LCCNN can detect from CT images the presence of lesions caused by COVID-19. This web-based app has a clear and simple interface, which is easy to use. With the help of this app, teachers can provide distance education and guide students clearly to understand the damage caused by COVID-19, which can increase interaction with students and stimulate their interest in learning.

## Introduction

 At the beginning of the COVID-19 pandemic outbreak in late 2019 [1–3], most people did not realize that it would be a global pandemic of great magnitude [4, 5]. The pandemic has spread worldwide and has already posed an enormous threat to people’s lives, with the number of confirmed cases still rising rapidly in all regions [6, 7]. Mutating viruses such as delta [8, 9] and omicron [10, 11] have put the pandemic situation under tension again and again. Fortunately, with the joint efforts of governments [12, 13], medical personnel [14, 15], and citizens [16, 17], the pandemic has been largely contained in many areas, and work and production are resuming in an orderly manner [18, 19].

COVID-19 is a lung disease caused by SARS-CoV-2 [20]. The virus is transmitted mainly by airborne droplets and contact [21] but can also be transmitted through objects or other surfaces [22]. Symptoms of infection with COVID-19 include fever, cough, malaise, and breathlessness [23]. Some patients may have more severe symptoms [24], such as pneumonia [25], lung infections [26], and loss of taste or smell [27, 28]. Severe infections can also cause infectious shock [29], sudden blood drops, a lack of oxygen to the body’s organs, and death [30]. People over 60 years of age with a smoking history and high blood pressure are relatively more likely to be infected [31, 32].

As a result of the pandemic, educational arrangements and requirements [33, 34] have been adjusted in schools around the world to meet the demands of the pandemic. Many courses taught offline have been changed to online distance education. In fact, for teachers [35, 36], transitioning from traditional face-to-face to online distance education is quite challenging [37]. Facing the unfamiliarity of the teaching methods, teachers need to constantly explore and improve their teaching approaches rather than simply copying the original teaching solutions. If teachers do not have adequate IT knowledge [38], it may not be easy to complete online teaching. For students [39, 40], during distance online education, students need to learn alone, which lacks the engagement of classroom lessons and can easily lead to fatigue. Distance education is limited because it cannot be taught face-to-face, so teachers must use rich and varied online resources to fill some teaching gaps [41]. If the teaching design is weak in interactivity for the teacher, this will result in a less engaging classroom for the students and a poorer overall outcome. This requires teachers to make more use of network resources, including images, audio, video and supplementary teaching platforms, to optimize the design of new teaching programs and improve teaching effectiveness in distance education.

Distance education often uses computer multimedia technology [42], computer network technology [43], and communication technology [44]. Distance education is a cross-regional mode of teaching and learning. There is no requirement for the location of students or teachers in this mode [45, 46]. The way information is transmitted, and the place of learning are flexible. It allows students to learn without the hindrance of time or space, thus allowing for personalized learning. The advantage of distance learning over face-to-face education is that distance learning offers students in poorer areas more opportunities to learn at a low cost.

Computer multimedia technology and network technology can provide teachers and students with a wide range and quality of teaching resources. Software, computers, mobile phones, and other hardware can support distance learning. For example, abstract theories can be visualized by drawing images with the powerful computing capabilities of computers. In the process of communication with people, computers enhance the sharing of resources, collecting and organizing information, and creation of databases. Database resources in text, audio, and video are increasingly used in education as new computer resources.

Currently, many studies are attempting to use computer network resources to help with distance education. Severino et al. (2021) [47] developed an online platform to help students up to second grade with basic learning. In her learning plan, she segmented multiple lessons according to the learning abilities of students of different ages, allowing for an improved user experience. Lowry et al. (2022) [48] developed a high-fidelity simulation platform. It combined with instructional videos and allows students to collaborate remotely to simulate laparoscopic surgery. The students made corrections and practice again and again based on feedback from the platform. The difference in performance between the students instructed by the teacher and those who practiced on the platform was small. There was a significant improvement in the students’ surgical performance after practicing with the platform. Zheng et al. (2022) [49] designed a simulation teaching resource for non-electrical students with the theme of safe electricity use. Students can practice the theory they have learnt by conducting realistic and more dangerous experiments on the platform. Hopefully, this will improve and compensate for the shortcomings of traditional teaching resources and teaching models. Lin (2022) [50] combined a variety of theoretical knowledge that students need to master to build a stable simulation platform. Students used this platform to simulate and practice foreign trade transactions. The technology in this learning platform can be changed according to the needs of teaching and learning to ensure that the content does not become outdated. Computer resources are also used in many areas, including transportation systems [51], emotion recognition [52], and action recognition [53].

We reviewed some advanced COVID-19 detection methods. Wang et al. (2020) [54] proposed a weakly supervised deep learning framework using pre-trained UNet for segmentation and feeding 3D images into a 3D deep neural network to obtain DeCovNet. Wu (2020) [55] combined wavelet Renyi entropy [56], feedforward neural network, and the 3SBBO algorithm. A better-performing WRE + 3SBBO was obtained. The paper [57] is similar to the idea in the paper [55]. It has three stages in the framework. El-kenawy et al. (2020) [57] proposed FSVC combining CNN, guided whale optimization algorithm [58]. The dataset used in the paper [59] was chest X-ray images. The proposed COVID-Net method has portability, availability, and rapid triaging. The 6 L-CNN [60] was a six-layer convolutional neural network that combines max pooling and batch normalization. The Adam algorithm [61] improved the detection of COVID-19 patients. The WE-CSO was based on wavelet entropy and cat swarm optimization [62] in the papers [60, 63]. The CNN in DLM [64] was trained to have a higher accuracy rate. The method GLCM-PSO proposed in the paper [65] combined grey-level cooccurrence matrix and PSO [66].

These approaches above still have some common disadvantages. These models are not explainable and have relatively low accuracy in recognizing whether the input image contains lesion regions or not. Although these models can assist in diagnosing COVID-19, they impose demanding requirements for the user. The user needs to have some medical knowledge. Also, their software interfaces are not easy to work on.

Our proposed lightweight, customized CNN (LCCNN)-based model and distance education app for COVID-19 recognition can solve the above problems. Our contributions are as follows:

- We propose an 8-layer lightweight, customized convolutional neural network.
- Five-channel data augmentation is proposed and used to help the model avoid overfitting.
- Our LCCNN model performs better than eight state-of-the-art models.
- Our LCCNN model costs fewer resources than six transfer learning models.
- Our LCCNN model is explainable for both radiologists and distance education users.
- Our LCCNN model is integrated into a distance education-based web app.

The rest of the paper is structured as follows. Section 2 describes the dataset we used in the course of our experiments. Section 3 describes the proposed 8-layer lightweight, customized convolutional neural network model (LCCNN). Section 4 discusses the results of the experiments conducted using our proposed model and the validation and compares the results with other existing state-of-the-art approaches. In Section 5, we conclude the research of this paper.

## Dataset

**Fig. 1:**
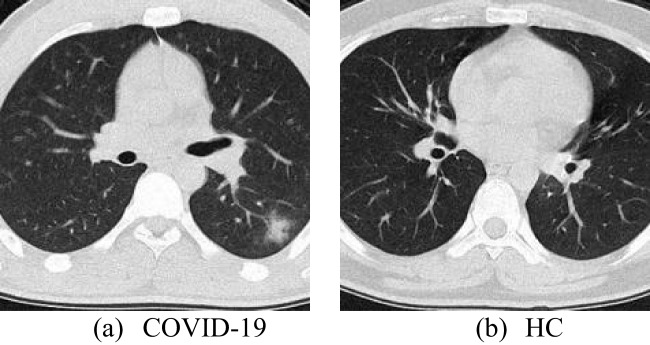
Samples from the dataset in this study

The dataset we used is from the paper [67]. A local hospital generated the dataset from 142 COVID patients (95 males and 47 females) and 142 healthy people (88 males and 54 females). After CT medical images are taken of both experimenters, the images are transferred to the medical image PACS. Two experienced physicians select clear and appropriate images for the dataset. The total dataset obtained consists of 640 images. Figure 1 shows two samples from the dataset this study used. The resolution of all images is 1024 × 1024.

## Methodology

Table 1 gives the abbreviation list in this study. Convolutional neural networks (CNNs) are designed based on the neural system that transmits signals in the human body. Neurons can respond to a part of the surrounding units in the range of the reach. CNN belongs to feed-forward neural networks and has outstanding performance in large-scale image processing.

**Table 1:** Abbreviation list

Abbreviation	Term
Acc	Accuracy
BN	Batch normalization layer
CAM	Class activation mapping
CNN	Convolutional neural network
Con	Convolutional layer
F1	F1 score
FDA	Five-channel data augmentation
FMI	Fowlkes–mallows index
Grad-CAM	Gradient-weighted class activation mapping
LCCNN	Lightweight customized convolutional neural network
MCC	Mathews correlation coefficient
MSD	Mean and standard deviation
Prc	Precision
ReLU	Rectified linear unit
RGB	Red, green, and blue
Sen	Sensitivity
Spc	Specificity

In the CNN, the signals enter the input layer, are treated with linear combinations and activation functions, then flow to the next layer. The signals are processed through each hidden layer and output to the output layer. Such signal delivery is a forward propagation process. The equation (Eq. 1) for forward propagation is as follows:

1 $${a}_{j}^{l}=\sigma \left(\sum\limits_{k=1}^{n}{w}_{jk}^{l}{a}_{k}^{l-1}+{b}_{j}^{l}\right),$$

where $n$ represents the number of feature maps in the last layer, i.e., $\left(l-1\right)$th layer. ${a}_{j}^{l}$ is the activation value of the $j$th neuron in the $l$th layer, which is also the output of the activation function. ${w}_{jk}^{l}$ denotes the weight of the $k$th neuron in the $\left(l-1\right)$th layer to the $j$th neuron in the $l$th layer. ${b}_{j}^{l}$ is the bias of the $j$th neuron in the $l$th layer. $\sigma$ represents the non-linear activation function.

Backpropagation is updating the weight of the parameters in the direction of the output layer to the input layer. The backpropagation is used for feedforward neural network parameter training, hoping to continuously iterate to optimize the model parameters based on the calculated error. In training the neural network, forward and backward propagation rely on each other. The usual cost function is the quadratic cost function. On this basis, assuming that the target data is one-dimensional, the equation (Eq. 2) [68] for calculating the cost $C$ between the output value and the actual value is:

2 $$C=\frac{1}{2 m}\sum _{i}^{m}{\left({y}_{oi}-{y}_{i}\right)}^{2}=\frac{1}{m}\sum _{i}^{m}{C}_{i},$$

where $m$ denotes the number of samples, ${y}_{oi}$ denotes the output of the $i$th sample, ${y}_{i}$ denotes the target data of the $i$th sample, and ${C}_{i}$ denotes the squared error of the output data and the target data of the $i$th sample.

The backpropagation process can be expressed by the equation (Eq. 3) [68]:

3 $$\begin{array}{c}\left\{\begin{array}{l}\delta {L}_{i}=\left({y}_{oi}-{y}_{i}\right){f}^{{\prime }}\left({z}_{i}^{L}\right)\\ \delta {l}_{i}={\left({W}^{l}\right)}^{T}\delta {\left(l+1\right)}_{i}{f}^{{\prime }}\left({z}_{i}^{l}\right)\\ \frac{\partial {C}_{i}}{{\partial W}^{l}}=\delta {\left(l+1\right)}_{i}{\left({a}_{i}^{l}\right)}^{T}\\ \frac{\partial C}{{\partial W}^{l}}=\sum\limits_{i}^{m}\frac{\partial {C}_{i}}{{\partial W}^{l}}\end{array} \right.,\end{array}$$

where ${z}_{i}^{l}$ denotes the input data at $l$th layer of the $i$th sample, ${a}_{i}^{l}$ denotes the output data at $l$th layer of the $i$th sample, ${W}^{l}$ denotes the weight of the $l$th layer. $f$ denotes the active function. $L$ denotes the output layer, and $\delta {l}_{i}$ denotes the error at $l$th layer of the $i$th sample. The superscript $T$ represents the matrix transpose.

### Convolutional layers

**Fig. 2:**
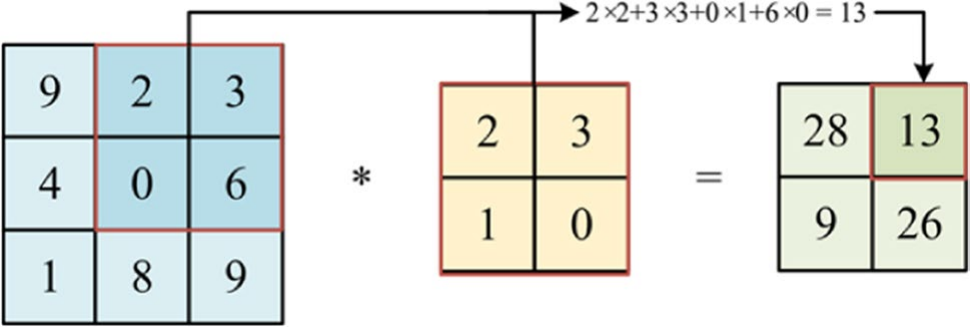
Diagram of a convolution calculation with one-channel input

The function of the convolutional layer is to extract features from the input data. The shallower convolutional layers extract local information, while deeper layers capture global information. A convolutional layer contains several convolutional kernels to create different feature maps. In a convolution layer, a kernel, known as a filter, slides over the input image according to the stride size. A convolutional kernel is a small matrix. During the convolution operation on an image, each value in the kernel is multiplied by the corresponding pixel value covered by the kernel. These multiplied values are then added together to obtain the value of the target pixel in the feature map. Figure 2 shows a schematic diagram of a single convolution operation. After one convolution, a new feature map of the image is generated.

**Fig. 3:**
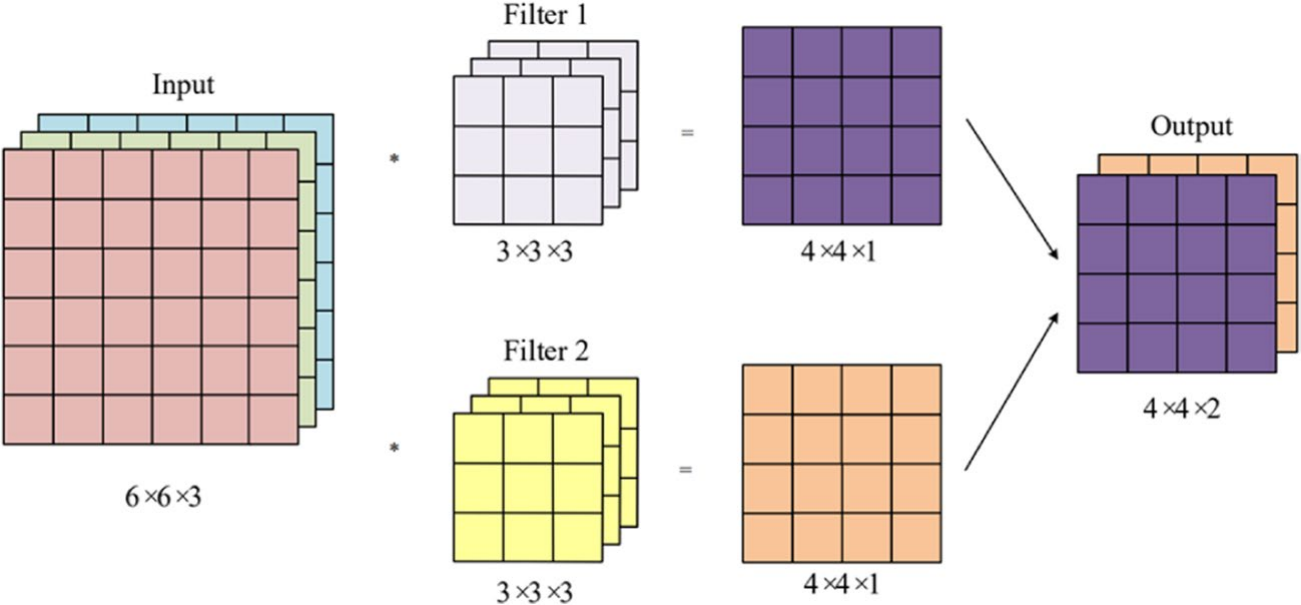
Illustration of an input image with three channels undergoing convolution

Single-layer convolution refers to a convolution operation using one convolution kernel. If the input image has three color channels (red, green, and blue), the convolution kernels must also have three channels. In three-channel convolution, the convolution kernels have length and width and a number of channels. The three-channel convolution performs convolution on each of the three channels of the image, resulting in the final output feature map. Figure 3 shows the process of a three-channel input image undergoing convolution with the stride step set to 1. After multiple convolutions, the output feature map can contain much information about the image, which can be used for image recognition tasks.

### Hyperparameters in convolutional layers

The hyperparameters in the convolution layer include the convolution kernel size, the stride size, and the padding way. The distance that the center of the convolution kernel moves once in two adjacent convolution operations is the stride size. Researchers can control the accuracy of feature extraction by adjusting the stride size. These hyperparameters determine the size of the output feature map of the convolution layer. The convolution kernel size can be specified as any value smaller than the size of the input image. The size of the convolution kernel determines the size of the receptive field. The receptive field represents the range of the convolution kernel’s effect. A larger convolution kernel has a wider receptive field. It can capture more complex features, while a smaller convolution kernel has a smaller receptive field and can only capture simple features. The equation (Eq. 4)  for calculating the receptive field size is as follows:

4 $${R}_{i}=\left({R}_{i+1}-1\right)\times {t}_{i}+{K}_{siz{e}_{i}},$$

where ${R}_{i}$ denotes the receptive field size of the $i$th layer, $i$ denotes the index of the current feature layer, $t$ is the stride size of the convolution, and ${K}_{{size}_{i}}$ is the size of the convolution kernel in this layer.

If the stride size is set small, there will be duplicate areas between adjacent step fields. If the stride size is set larger, there will not be duplicate areas between adjacent step fields, but maybe parts that are not covered, causing the information of the original image to be missed. Typically, the convolution kernel is a square with an odd number of side lengths and is located using the center.

**Fig. 4:**
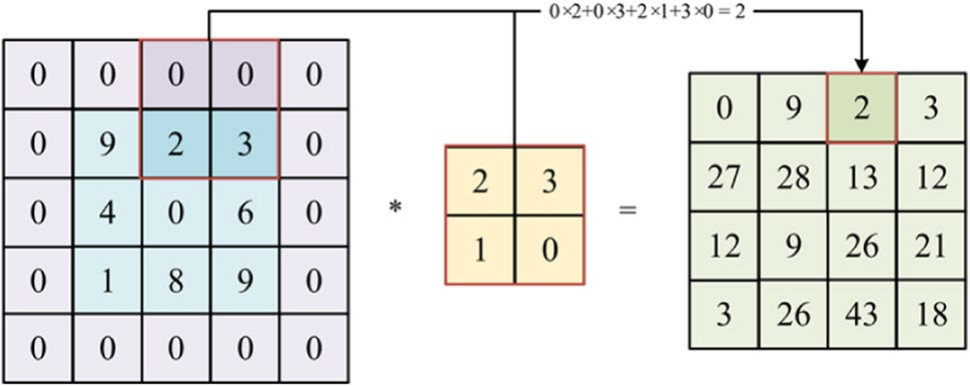
A diagram of the convolution calculation after padding

As demonstrated in Fig. 2, the shape of its output is reduced after the convolution operation is performed on the image. Scholars apply padding operations to preserve the image’s size while still undergoing convolution. Padding involves adding additional rows or columns, usually filled with zeros, around the edges of the input. This can be visualized in Fig. 4, where the image’s shape remains constant after the convolution operation. By padding the image and then convolving it to extract features, we can focus on the features at the edges, making them as important as the features in the middle.

In addition to the common single-channel convolution and multi-channel convolution, researchers have also attempted convolution by using multi-dimensional data. 3D CNN models use 3D images as input. The structure of a 3D CNN is similar to a standard 2D CNN model. In this case, the convolution layer uses 3D kernels for filtering. In a 2D CNN, the convolution kernel moves in two directions, while in a 3D convolution, it moves in three directions. 3D convolution requires more computational power and memory space for storing parameters and feature space than 2D CNN.

### Pooling layers

Pooling layers are usually located in the middle of successive convolutional layers. Unlike convolutional layers, pooling layers do not have parameters to learn. Common pooling operations are average pooling, max pooling, and random pooling.

Average pooling aims to keep more of the image background information. Max pooling is used to reduce the bias in the mean value of the estimate caused by parameter errors in the convolution layer to preserve more of the texture information.

**Fig. 5:**
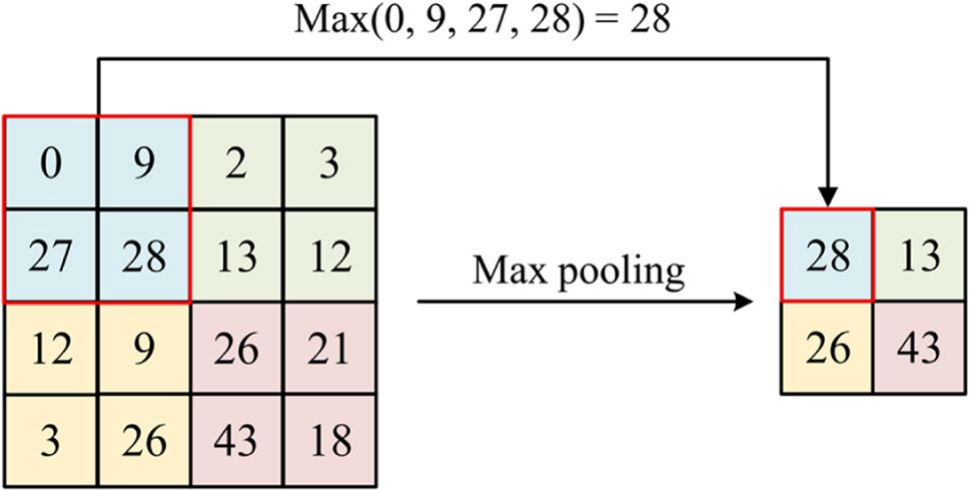
Illustration of a max pooling operation

Using max pooling as the example, we first set up a sliding window and obtain the largest value from the image corresponding to the window as the output feature’s corresponding position value. This window is then slid to the next position in the general order of the set step from left to right, top to bottom. The example in the diagram shows a 2 × 2 window with a stride set of 2. The initial window is in the blue area of the diagram. The maximum value of 28 is taken from the window and passed to the following feature map, as shown in Fig. 5.

Average pooling mainly reduces the data size by taking the average value in each neighborhood, thus reducing computational costs. On the other hand, max pooling reduces the data size by taking the maximum value in each neighborhood, thus preserving important features. This study chooses max pooling. The max-pooling operation only extracts the maximum value in each rectangular region, the part with the strongest response, into the next layer. Using max-pooling means that features can be identified no matter where in the image they are located. With many images containing objects on the input, a model with good performance can be obtained.

When pooling is performed, the output results are less affected, even if there are minor deviations in the input data. The number of channels of input and output data will not change after the pooling. The number of parameters that need to be computed by the model is reduced, redundant information is cut, and the network’s complexity is decreased.

### ReLU activation function

The activation function, an improvement proposed to solve linearly indistinguishable problems, maps a neuron’s input to the output side. In solving real problems, the data distribution is overwhelmingly non-linear, and it isn’t easy to rely solely on using linear neural network computations to solve them. It is, therefore, necessary to incorporate non-linear activation functions to enhance the learning capability of the network to make the neural network applicable to a wider range of models.

Various activation functions are available, which correspond to different properties and are suitable for different situations. The Sigmoid activation function is the first activation function used in neural networks and is often used in binary classification problems. Sigmoid activation functions predict results clearly but are prone to gradient disappearance. The Sigmoid activation function is not 0-centered, and convergence is slow when the number of layers is too much, making deep training impossible.

**Fig. 6:**
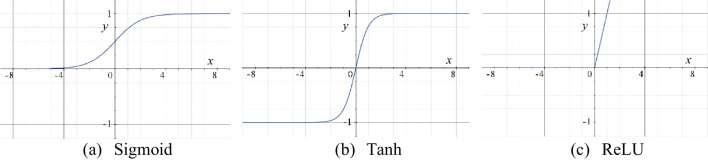
Three more common activation functions (Coordinate axis ratio is $x:y=4:1$)

The Tanh function speeds up convergence based on the Sigmoid activation function but requires more computation for both forward and backward propagation. The ReLU activation function is prevalent in deep learning. It doesn’t have the gradient disappearance problem. Its speed of convergence and computational speed is fast. Its generality allows it to be used in several studies with a wide range of uses. The equations (Eq. 5) of Sigmoid, Tanh, and ReLU function activation, in turn, are as follows, and the function images are shown in Fig. 6.

5 $$\begin{array}{l}\left\{\begin{array}{l}\text{S}\text{i}\text{g}\text{m}\text{o}\text{i}\text{d}\left(x\right)=\frac{1}{1+{e}^{-x}}\\ \text{T}\text{a}\text{n}\text{h}\left(x\right)=\frac{{e}^{x}-{e}^{-x}}{{e}^{x}+{e}^{-x}}\\ \text{R}\text{e}\text{L}\text{U}\left(x\right)=\text{max}\left(0,x\right)\end{array}\right.\end{array}$$

### Proposed LCCNN model

The more important layers in our proposed model are the six convolutional layers and the two fully connected layers. Our input image only has a single channel. The input layer size is 256 × 256 × 1. Con1 is a convolutional layer using 16 feature maps of size 3 × 3 × 1 with a stride size of 2. After the max pooling operation with the window size of 2 × 2, the output size is 64 × 64 × 16. Con2 is a convolutional layer using 32 feature maps of size 3 × 3 × 16 with a stride size of 1. After the max pooling operation with the window size of 2 × 2, the output size is 32 × 32 × 32. Con3 is a convolutional layer using 64 feature maps of size 3 × 3 × 32 with a stride size of 1. After the max pooling operation with the window size of 2 × 2, the output size is 16 × 16 × 64.

Con4 is a convolutional layer using 64 feature maps of size 3 × 3 × 64 with a stride size of 1. After the max pooling operation with the window size of 2 × 2, the output size is 8 × 8 × 64. Con5 is a convolutional layer using 128 feature maps of size 3 × 3 × 64 with a stride size of 1. The output size is 8 × 8 × 128. Con6 is a convolutional layer using 128 feature maps of size 3 × 3 × 128 with a stride size of 1. The output size is 8 × 8 × 128. The two fully connected layers are in size 200 × 8192 and 2 × 200, the details of the model are shown in Table 2.

**Table 2:** Structure of the proposed 8-layer LCCNN model

Layer	BN Layer	Hyperparameters	Size of Learnable Parameters	Output Size
Input				256 × 256 × 1
Con1		16 3 × 3 × 1, S = 2, P = 2	160	64 × 64 × 16
	BN1		32	
Con2		32 3 × 3 × 16, S = 1, P = 2	4640	32 × 32 × 32
	BN2		64	
Con3		64 3 × 3 × 32, S = 1, P = 2	18,496	16 × 16 × 64
	BN3		128	
Con4		64 3 × 3 × 64, S = 1, P = 2	36,928	8 × 8 × 64
	BN4		128	
Con5		128 3 × 3 × 64, S = 1	73,856	8 × 8 × 128
	BN5		256	
Con6		128 3 × 3 × 128, S = 1	147,584	8 × 8 × 128
	BN6		256	
FCL1		200 × 8192	1,638,600	200
FCL2		2 × 200	402	2

BN = Batch Normalization, S = Stride, P = Pooling filter size

### Explainability of LCCNN

Common methods used to interpret CNN models are class activation mapping (CAM) and Gradient-weighted class activation mapping (Grad-CAM). Class activation mapping uses different colors to indicate the regions associated with the target class.

The Grad-CAM improves on the CAM by looking to derive weights from gradients. Grad-CAM is a weighted sum of feature maps, followed by a ReLU operator. It (Eqs. 6, 7) can be calculated in Ref. [69]:

6 $${\alpha }_{k}^{c}=\frac{1}{Z}\sum\limits_{i}\sum\limits_{j}\frac{\partial {y}^{c}}{\partial {A}_{ij}^{k}}$$

7 $${L}_{Grad-CAM}^{c}=\text{ReLU}\left(\sum\limits_{k}{\alpha }_{k}^{c}{A}^{k}\right),$$

${\alpha }_{k}^{c}$ denotes the weight obtained after the gradient of the return flow has been globally averaged for a target class $c$. ${A}_{ij}^{k}$ represents the $k$th channel of feature map $A$, where the spatial index is ($i,j$). ${y}^{c}$ represents the score predicted by the network for class $c$ before the softmax. $Z$ is the spatial resolution of the feature map, which equals the width of the feature layer multiplied by its height. $\partial {y}^{c}/\partial {A}_{ij}^{k}$ represents the gradient via backpropagation.

CAM requires the CNN based on global average pooling architecture, making the method difficult to apply to most CNN architectures. The structure of the CNN does not affect the Grad-CAM operation. Grad-CAM uses the gradient information flowing into the last convolutional layer of the CNN to understand the importance of each neuron to the decision.

### Cross validation

Cross-validation is one of the essential methods used by scientists when performing statistical analysis. It is often necessary for the practice to verify a model’s stability and generalization ability to a new dataset. It is needed to ensure that the model obtained from the dataset has most of the correct information about the dataset without containing too much noise. In other words, the models’ bias and variance are small.

In the process of $K$-fold cross-validation, the dataset must be divided into almost equal K smaller datasets. The process of extracting different smaller folds as a test set and calculating the average can be represented by the equation (Eq. 8):

8 $$e=\frac{1}{K}\sum\limits_{i=1}^{K}{E}_{i},$$

${E}_{i}$ represents the output result of the $i$ th test. $e$ represents the average of the results of the $K$ tests.

**Fig. 7:**
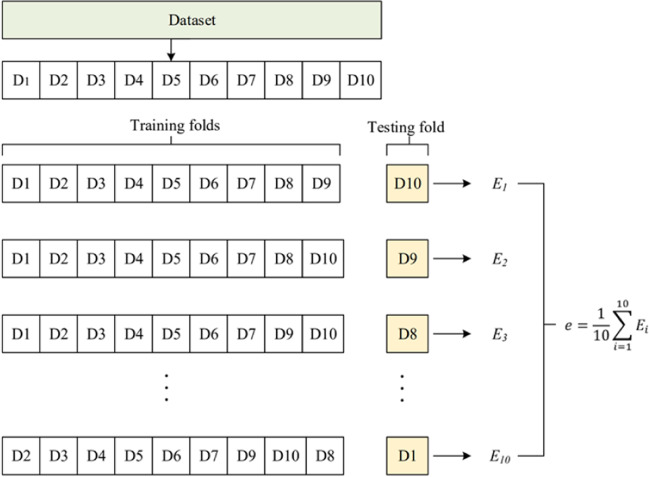
Illustration of a 10 × 10-fold cross-validation

The obtained dataset is divided into training and testing sets in cross-validation. As shown in Fig. 7, we divide the data set into ten folds (D1, D2, …, D10) and then perform ten tests, changing a different testing set each time. In the end, we get ten models’ results and take the average as a result. ${E}_{i}$ denotes the output result of the $i$th test and $e$ denotes the average of the results of the $i$ tests.

### Proposed five-channel data augmentation

Scholars struggle to have enough data to complete their tasks in many practical projects. We can expand our existing data by data augmentation. Data augmentation allows the model to classify more stable and helps the model recognize images in different conditions. Data augmentation allows existing data sets to be used to greater effect by increasing the number of times the model is trained.

Several common data augmentation methods include adding noise, cropping, flipping, rotation, scaling, and brightness. We hope that the proposed FDA method will be easy to achieve. Geometry-based data augmentation (GDA) methods are easy to perform. Unfortunately, it is often necessary to observe the data generated by the GDA method manually to ensure whether the image labels need to be redefined. In addition to hoping that the data augmentation approaches would be easy to realize, we also wanted to enhance the model’s generalization ability. By noise-injection data augmentation (NIDA), the robustness and generalization of the model can be improved.

Based on the above reasoning, we have chosen five methods of data augmentation from GDA and NIDA: translation, scaling, rotation, horizontal shear, and Gaussian noise injection, with the help of combining GDA and NIDA. The five-channel data augmentation (FDA) proposed consists of these five methods, as shown in Table 3.

**Table 3:** Five methods in FDA

Type	Method	Definition
I	Translation	Translation involves moving the image along the $x$ or $y$ direction or both. If the data in the dataset are in different sizes, it is important to normalize the data using shear or scaling, which may produce distortion.
II	Scaling	Scaling is the act of enlarging or diminishing a linear transformation of an image.
III	Rotation	Rotation means the act of turning around a center or an axis.
IV	Shear	The shear operation is to shear an image from both corners with some probability in the $x$ and y directions.
V	Noise Injection	Gaussian noise injection adds Gaussian noise to the original data, reducing the model’s risk of learning irrelevant but high-frequency features.

**Fig. 8:**
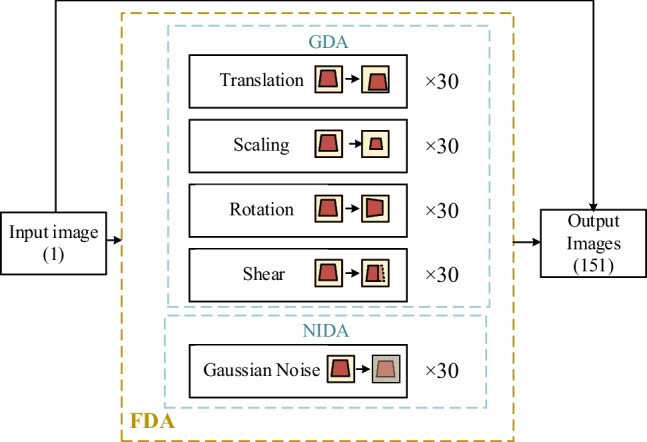
Flowchart of the proposed FDA, which combines GDA and NIDA

Figure 8 shows the flowchart of the FDA. In the experiment, an input image is processed through multiple data augmentations. Each data augmentation method outputs 30 images.

## Experiment results and discussions

### Five-channel data augmentation

**Fig. 9:**
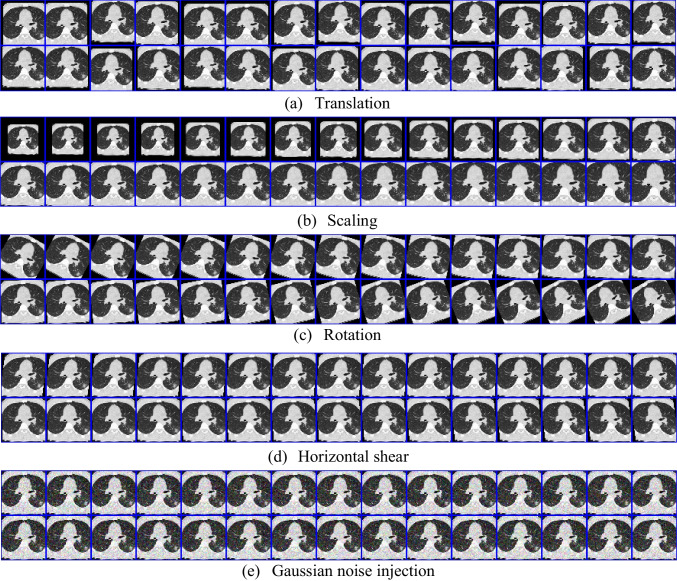
Result of FDA

The results of the FDA are shown in Fig. 9, and we can observe five results: translation, scaling, rotation, horizontal shear, and Gaussian noise injection. Figure 9 shows that FDA can increase the variety of the dataset and compensate for the disadvantage of having fewer data in the dataset.

### The results of the LCCNN

We obtained Table 4 after validating the performance of the LCCNN using 10$\times$10-fold cross-validation. The results of each indicator are expressed in terms of mean and standard deviation (MSD). The sensitivity is 91.44 ± 1.78, the specificity is 92.12 ± 1.37, the precision is 92.10 ± 1.15, the accuracy is 91.78 ± 0.44, the F1 score is 91.75 ± 0.52, the MCC is 83.60 ± 0.88, and the FMI is 91.76 ± 0.52. Table 4 shows that the experiment performed well, with the obtained performance indicators being less far from the mean and with smaller standard deviations. The results of each run had slight differences and performed flatly. The results fluctuated less for different subsets as the testing set. Sensitivity fluctuated the most, reaching 1.78. Among the multiple metrics, the accuracy values fluctuated the least, and the mean values were high, indicating that the LCCNN classifier was effective.

**Table 4:** Results of $10\times 10$-fold cross-validation

Run	Sen	Spc	Prc	Acc	F1	MCC	FMI
1	90.94	92.81	92.68	91.88	91.80	83.76	91.80
2	90.94	92.19	92.09	91.56	91.51	83.13	91.51
3	92.19	91.25	91.33	91.72	91.76	83.44	91.76
4	89.69	92.81	92.58	91.25	91.11	82.54	91.12
5	93.44	90.00	90.33	91.72	91.86	83.49	91.87
6	93.75	90.94	91.19	92.34	92.45	84.72	92.46
7	93.75	90.62	90.91	92.19	92.31	84.42	92.32
8	88.75	93.12	92.81	90.94	90.73	81.95	90.76
9	90.94	93.12	92.97	92.03	91.94	84.08	91.95
10	90.00	94.38	94.12	92.19	92.01	84.46	92.04
MSD	91.44 ± 1.78	92.12 ± 1.37	92.10 ± 1.15	91.78 ± 0.44	91.75 ± 0.52	83.60 ± 0.88	91.76 ± 0.52

### Convergence plot

**Fig. 10:**
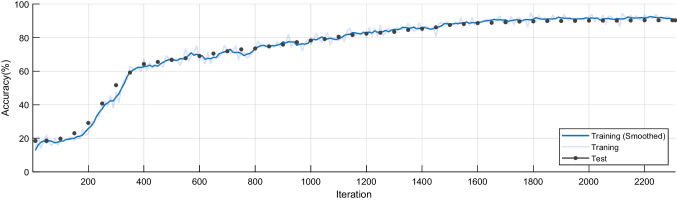
Convergence plot of the accuracy against iterations

Accuracy is one of the essential standards for evaluating the performance of a model. Figure 10 shows the process of accuracy change in the iterations. From Fig. 10, the light blue line shows the actual training results, the dark blue line shows the trend of the smoothed training results, and the black dots indicate the test results. As can be seen from the graph, the accuracy rate increases but tends to rise more slowly for training rounds less than the 200th iteration. The accuracy rate rises fast in training rounds in the 200th-400th iteration. The curve changes more slowly in the 400th-1800th iteration. In fact, after the 1800th iteration, we can see that the total performance has leveled off and remains at a high value.

### Structure comparison

Although many studies are increasingly inclined to investigate deeper CNN models, the fact is that it is not beneficial to have too many layers in a CNN model. With increasing layers, more problems need to be solved, including computational power, gradient, activation function, etc., and even new problems may appear. The improvement in effectiveness is not significant after the number of layers is increased to a certain number. In building the model, we hope to set the most suitable number of layers to help LCCNN achieve the best results.

For this purpose, we set up models with different numbers of layers to test them separately. The final number of layers of LCCNN was determined by comparing the data obtained from the experiments. Table 5 shows the data obtained by running 10$\times$10-fold cross-validation with the separate 7-layer and 9-layer model settings. The comparative bar chart is based on Tables 4 and 5.

**Table 5:** Results for 10-fold cross-validation of the 7-layer and 9-layer experimental models

Run	Sen		Spc		Prc		Acc		F1		MCC		FMI	
	*n* = 7	*n* = 9	*n* = 7	*n* = 9	*n* = 7	*n* = 9	*n* = 7	*n* = 9	*n* = 7	*n* = 9	*n* = 7	*n* = 9	*n* = 7	*n* = 9
1	90.00	92.50	92.19	92.19	92.01	92.21	91.09	92.34	91.00	92.36	82.21	84.69	91.00	92.36
2	89.69	87.81	90.62	93.12	90.54	92.74	90.16	90.47	90.11	90.21	80.32	81.05	90.11	90.24
3	92.50	91.88	91.25	91.88	91.36	91.88	91.88	91.88	91.93	91.88	83.76	83.75	91.93	91.88
4	91.56	90.94	90.62	90.62	90.71	90.65	91.09	90.78	91.14	90.80	82.19	81.56	91.14	90.8
5	90.62	89.06	88.75	91.56	88.96	91.35	89.69	90.31	89.78	90.19	79.39	80.65	89.79	90.20
6	91.25	91.25	92.19	91.88	92.11	91.82	91.72	91.56	91.68	91.54	83.44	83.13	91.68	91.54
7	90.94	91.25	89.69	91.25	89.81	91.25	90.31	91.25	90.37	91.25	80.63	82.50	90.37	91.25
8	89.38	90.94	90.00	90.00	89.94	90.09	89.69	90.47	89.66	90.51	79.38	80.94	89.66	90.51
9	86.88	91.56	90.00	91.56	89.68	91.56	88.44	91.56	88.25	91.56	76.91	83.12	88.27	91.56
10	90.00	89.38	89.69	92.19	89.72	91.96	89.84	90.78	89.86	90.65	79.69	81.59	89.86	90.66
MSD	90.28	90.66	90.50	91.62	90.48	91.55	90.39	91.14	90.38	91.09	80.79	82.30	90.38	91.10
	± 1.53	± 1.45	± 1.11	± 0.87	± 1.06	± 0.76	± 1.06	± 0.68	± 1.09	± 0.74	± 2.12	± 1.35	± 1.09	± 0.73

*n* stands for the number of layers

**Fig. 11:**
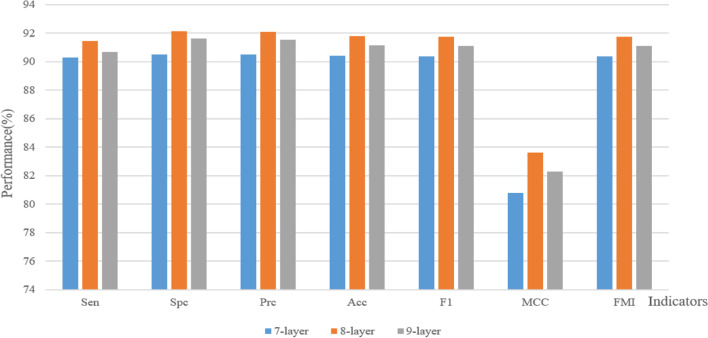
Comparison of mean values of various indicators between different network structures with different numbers of layers

By observing Fig. 11, we can see that the performance of the 8-layer model is improved over that of the 7-layer model. The performance of the 8-layer model is slightly better than that of the 7-layer or 9-layer model. However, there is no improvement when the number of layers is increased over the 8-layer model, but rather a decrease in performance. In the end, we concluded that the best results were obtained when the number of layers was 8.

### Comparison of state-of-the-art approaches

To understand the level of the LCCNN model, we selected eight state-of-the-art approaches to compare with our proposed 10$\times$10-fold cross-validation metrics for LCCNN. The advanced methods include DeCovNet [54], WRE + 3SBBO [55], FSVC [57], COVID-Net [59], 6 L-CNN [60], WE-CSO [63], DLM [64], GLCM-PSO [65]. Indicators include Sen, Spc, Prc, Acc, F1, MCC, and FMI.

**Fig. 12:**
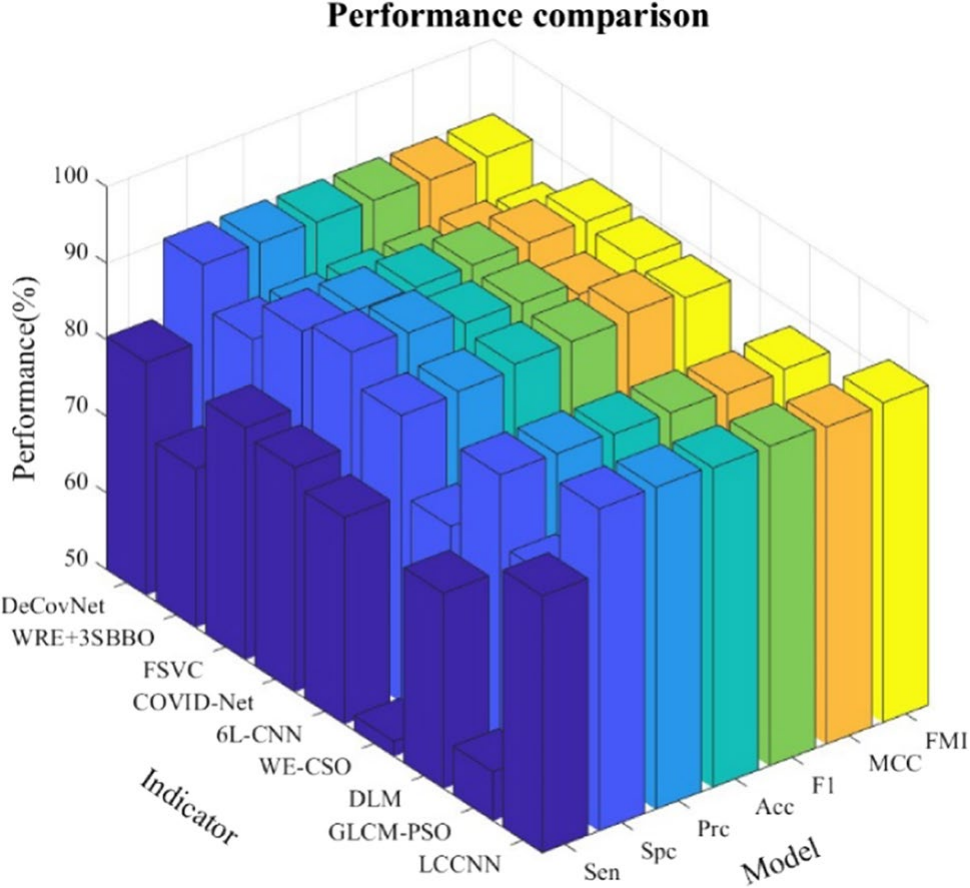
Bar plot of comparison with state-of-the-art models

Details are listed in Table 6. we plotted Fig. 12, and the data used do not include fluctuation data. The bar charts help us to see the comparison results more visually. The sensitivity of LCCNN is slightly higher than that of FSVC, but the fluctuations in the data are also relatively large. In terms of accuracy, which is more critical, LCCNN is less volatile and more accurate than DeCovNet and is more stable.

**Table 6:** Performance comparison with state-of-the-art models

Model	Sen	Spc	Prc	Acc	F1	MCC	FMI
DeCovNet [54]	90.03 ± 1.22	90.34 ± 1.25	90.33 ± 1.07	90.19 ± 0.68	90.17 ± 0.69	80.39 ± 1.35	90.18 ± 0.68
WRE + 3SBBO [55]	85.94 ± 1.68	84.75 ± 2.42	84.96 ± 2.16	85.34 ± 1.81	85.44 ± 1.74	70.71 ± 3.61	85.44 ± 1.73
FSVC [57]	90.25 ± 1.27	90.03 ± 0.80	90.06 ± 0.72	90.14 ± 0.70	90.15 ± 0.73	80.29 ± 1.41	90.15 ± 0.74
COVID-Net [59]	87.66 ± 2.39	91.53 ± 1.42	91.21 ± 1.29	89.59 ± 1.16	89.38 ± 1.28	79.28 ± 2.27	89.41 ± 1.26
6 L-CNN [60]	89.47 ± 1.50	87.47 ± 2.11	87.75 ± 1.76	88.47 ± 1.05	88.59 ± 0.99	76.98 ± 2.09	88.60 ± 0.99
WE-CSO [63]	74.75 ± 2.02	77.19 ± 1.41	76.63 ± 0.89	75.97 ± 0.80	75.66 ± 1.04	51.97 ± 1.56	75.68 ± 1.02
DLM [64]	87.37 ± 1.51	88.12 ± 1.94	88.06 ± 1.75	87.75 ± 1.31	87.71 ± 1.29	75.52 ± 2.62	87.71 ± 1.29
GLCM-PSO [65]	77.36 ± 2.47	78.99 ± 2.31	78.69 ± 1.55	78.18 ± 0.86	77.99 ± 1.06	56.41 ± 1.73	78.01 ± 1.05
LCCNN (Ours)	**91.44** **± 1.78**	**92.12** **± 1.37**	**92.10** **± 1.15**	**91.78** **± 0.44**	**91.75** **± 0.52**	**83.60** **± 0.88**	**91.76** **± 0.52**

Bold means the best

### Network complexity comparison

The designers of early classical CNNs such as AlexNet, VGG16, GoogleNet, ResNet, and MobileNet focused on improving the accuracy of their respective classifications. Designers rarely consider the number of parameters and memory storage.

**Fig. 13:**
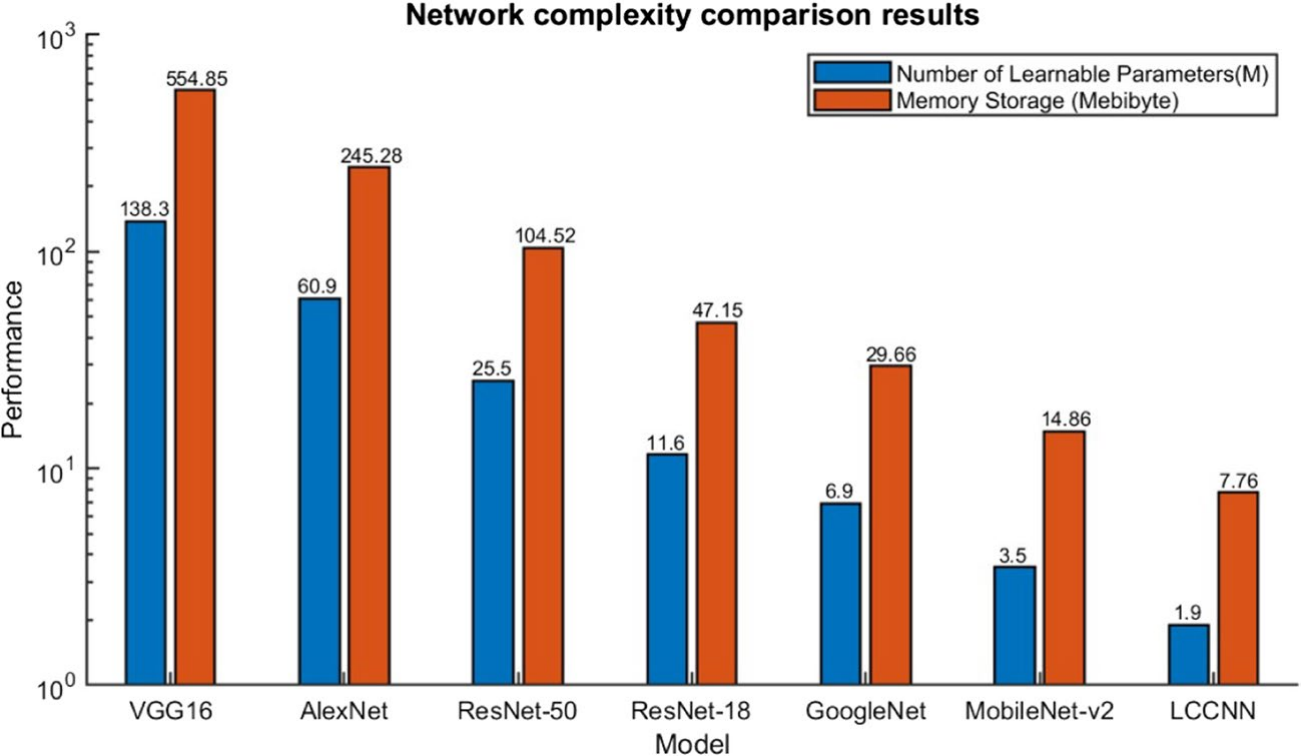
Network complexity comparison

As shown in Table 7; Fig. 13, this study provides two metrics, the number of learnable parameters and memory storage. These two metrics are used to indicate the complexity of the network structure. Our proposed LCCNN model has the smallest number of learnable parameters and memory storage compared with other classical models. Remarkably, the number of learnable parameters in LCCNN is only 1.3% of that in VGG16.

**Table 7:** Network complexity comparison results

Model	Number of Learnable Parameters$\downarrow$	Memory Storage (Byte)$\downarrow$
VGG16	138.3 M	554.85 M
AlexNet	60.9 M	245.28 M
ResNet-50	25.5 M	104.52 M
ResNet-18	11.6 M	47.15 M
GoogleNet	6.9 M	29.66 M
MobileNet-v2	3.5 M	14.86 M
LCCNN (Ours)	**1.9 M**	**7.76 M**

Bold means the best, $\downarrow$ means the smaller the better

### Distance education app

In offline teaching, it is often difficult for teachers to clearly explain abstract concepts or knowledge. Therefore, the use of a remote education application is important. For this purpose, we have designed a web-based app based on LCCNN to diagnose COVID-19 CT images. This app assists teachers in providing detailed explanations and helps students better understand the course content.

**Fig. 14:**
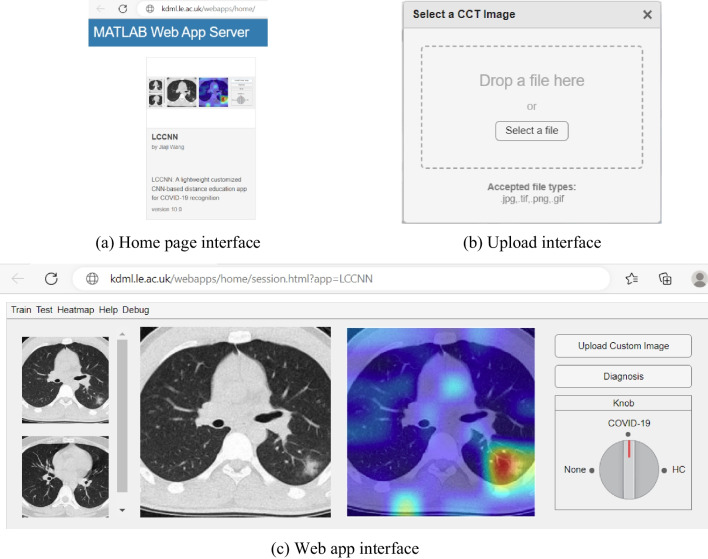
The interactive interfaces of the app

Figure 14(a) shows the home page of our app. The user can access the web app by clicking on the graphical icon and start using the app.

In the web app interface, as shown in Fig. 14(c), the leftmost images are those waiting to be recognized. In the middle are two larger CT images, the left of which is being classified, and the right is the heat map generated from the left-side CT image.

There are two buttons on the rightmost side of the interface. Clicking on the topmost ‘upload custom image’ button allows you to upload a new unclassified image, and the pop-out window is shown in Fig. 14(b). Once the upload is complete, click the ‘diagnosis’ button to start the diagnosis. The label pointed by the red Knob shows the result of the diagnosis.

### Explainability

**Fig. 15:**
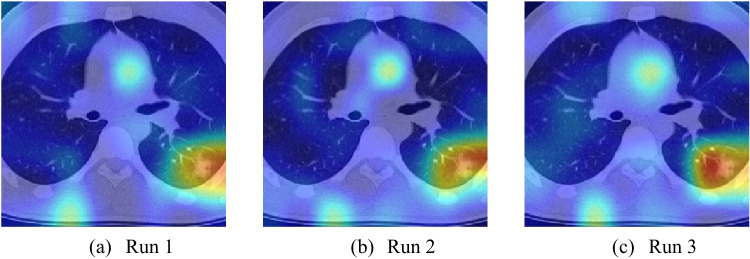
Images obtained using Grad-CAM during the 10-fold cross-validation process of LCCNN

As the app is aimed at helping teachers to show students the effects of COVID-19 on the lungs, our model needs to be explainable. Figure 15 shows the heat map obtained by our LCCNN model. From left to right in Fig. 15, the heat maps are produced from the first to the third runs.

Observing the positions and areas of the different colors in the images, we can find that though the run index may vary, the model remains accurate in identifying problematic areas of the images. The red highlighted area in Run 1 is located to the right of the lesion area. The red area in Run 2 is closer to the actual lesion area than in Run 1. In Run 3, the red highlighted area almost coincides with the actual lesion area.

## Conclusions

In this paper, we design a distance app based on an 8-layered LCCNN for COVID-19 recognition. We hope this app will help teachers demonstrate the damage caused by COVID-19 in the lungs to their students in the classroom.

The distance education app provides a more visual way of teaching and taking advantage of multimedia equipment to give students a better understanding of the disease and the importance of protection. The core component of the app is an 8-layered LCCNN for COVID-19 recognition named LCCNN. It can classify the input lung CT images more accurately and quickly identify the CT images containing the lung’s diseased part. For data augmentation, we propose the five-channel data augmentation. FDA is easy to operate and can improve the robustness of the model. We evaluated its effectiveness through 10$\times$10-fold cross-validation. The test results were then compared with eight state-of-the-art image classification methods: DeCovNet, WRE + 3SBBO, FSVC, COVID-Net, 6 L-CNN, WE-CSO, DLM, and GLCM-PSO. The comparison results show that the LCCNN model has improved in each evaluation metric compared to the above eight state-of-the-art methods. The differences in classification accuracy obtained from testing on different test sets were small.

Although our proposed 8-layered LCCNN for COVID-19 recognition is a lightweight deep neural network model, we will develop a standalone app that can be installed and run on any mobile phone. Apart from that, although our web-based app works correctly, the interface is relatively clean, and we will beautify it in the future. For the LCCNN model, we will look for other publicly-available datasets to train on and improve its recognition accuracy.

## Data Availability

The datasets analyzed during the current study are available from the corresponding author upon reasonable request.
